# C/EBP homologous protein drives pro-catabolic responses in chondrocytes

**DOI:** 10.1186/ar4415

**Published:** 2013-12-19

**Authors:** Matt Husa, Freyr Petursson, Martin Lotz, Robert Terkeltaub, Ru Liu-Bryan

**Affiliations:** 1 Department of Medicine, The Ohio State University, Columbus, OH, USA; 2 St Louis University School of Medicine, St Louis, MO, USA; 3 Department of Molecular and Experimental Medicine, The Scripps Research Institute, La Jolla, CA, USA; 4 Department of Medicine, VA Medical Center, University of California San Diego, San Diego, CA, USA; 5 Veterans Affairs Healthcare System, University of California San Diego, 111K, 3350 La Jolla Village Drive, San Diego, CA 92161, USA

## Abstract

**Introduction:**

Excess C/EBP homologous protein (CHOP) expression is one feature of the unfolded protein response (UPR) to endoplasmic reticulum (ER) stress. Here, we focused on CHOP expression and function in chondrocytes.

**Methods:**

We studied human knee osteoarthritis (OA) cartilage, bovine chondrocytes cultured in alginate and subjected to sub-lethal biomechanical injury, and knee chondrocytes of human autopsy donors. We performed siRNA knockdown and transfection.

**Results:**

UPR activation was increased in human knee OA cartilage *in situ*, and in biomechanically injured cultured chondrocytes *in vitro*. In normal human chondrocytes, CHOP “gain of function” sensitized chondrocytes to IL-1β induced nitric oxide (NO) and matrix metalloproteinase (MMP)-3 release without inducing these responses by itself. Excess CHOP expression, by itself, induced superoxide production and apoptosis. Conversely, siRNA knockdown of CHOP and the UPR-specific mediator X-box binding protein (XBP1) inhibited NO release by >80% (*P* <0.0005) in response to IL-1β, and blunted MMP-3 release, whereas there were only minimal effects of the UPR mediator GRP78 on these responses. The anti-inflammatory metabolic “super-regulator” AMP kinase (AMPK) is known to limit UPR activation in vascular muscle cells. Here, CHOP supported the capacity of IL-1β to suppress AMPK activity in chondrocytes. We also observed that inhibition of AMPK activity promoted an increase in chondrocyte CHOP expression. Conversely, pharmacologic activation of AMPK by 5-Aminoimidazole-4-carboxamide ribonucleotide (AICAR) blunted chondrocyte CHOP expression in response to biomechanical injury.

**Conclusions:**

Biomechanical injury and IL-1 signaling stimulate UPR activation in chondrocytes. CHOP mediates chondrocyte catabolic and apoptotic responses to IL-1β, and does so partly by inhibiting AMPK activity. Conversely, development of excess CHOP activity is limited by AMPK activity in chondrocytes. Our findings suggest a mechanism for potential chondroprotection by AICAR and other AMPK activators. The work is of translational relevance for OA, since several drugs that activate AMPK are already in the clinic for arthritis (for example, allosteric AMPK activators sodium salicylate and high dose aspirin, and methotrexate, which activates AMPK by generating AICAR).

## Introduction

Chondrocyte stress responses to biomechanical injury and joint inflammation, and associated changes in differentiation and function, provide a foundation upon which cartilage injury and osteoarthritis (OA) can be triggered and accelerated [1]. Fundamental proteostasis responses by which cells normally resolve stress include the unfolded protein response (UPR), which restores equilibrium to the stressed endoplasmic reticulum (ER) via a reprogrammed proteome, rich in chaperones and protein-folding catalysts [2,3]. The UPR also regulates oxidative stress responses, inflammation, and cell fate [4].

In the UPR, three signaling/proteolytic cascades are triggered by dissociation of GRP78, a chaperone for misfolded proteins in the ER lumen, from the ER transmembrane proteins pancreatic ER (PERK), inositol-requiring enzyme 1 (IRE1), and activating transcription factor 6 (ATF6) [2-4]. GRP78 normally dampens the UPR and is anti-apoptotic [4]. The PERK pathway is turned on by IL-1β and nitric oxide (NO), is known to be activated in OA chondrocytes, and slows protein translation by phosphorylating eukaryotic initiation factor 2A (eIF2A), thereby relieving pressure on the ER [4,5]. IRE1, following GRP78 release, mediates UPR-specific x-box binding protein (XBP1) alternative mRNA splicing, generating the potent transcriptional activator spliced XBP1 (XBP1s), which is approximately 130 amino acids longer than unspliced XBP1 (XBP1u), and has a much longer half-life [6]. XBP1s promotes ER degradation (ERAD) of aberrant proteins, and can exert either pro- or anti-inflammatory effects [7,8].

The PERK pathway plays a major role in promoting expression of C/EBP binding protein (CHOP) in the UPR [2,4]. CHOP constitutively functions to resolve the UPR, restoring protein synthesis by acting on the phosphatase growth arrest and DNA damage-inducible protein 34 (GADD34), which dephosphorylates eIF2A [4,9]. CHOP normally has a short half-life [9,10]. However, prolonged or excess CHOP expression drives apoptosis by loading an already stressed ER with more protein, and via other CHOP transcriptional effects on pro- and anti-apoptotic factors and by hyper-oxidizing the ER [4,9].

Triggering of growth-plate cartilage pathology by loading the ER with misfolded transgenic mutant proteins (type X collagen, matrilin-3) first illustrated chondral effects of excess UPR activation [11,12]. In OA cartilage, increased XBP1 activation and expression of GRP78 and CHOP are evidence of heightened ER stress *in situ*[13,14]. Cultured OA chondrocytes demonstrate evidence of PERK module activation [15-17]. This includes expression of TRB3, an Akt inhibitor that inhibits the capacity of insulin-like growth factor-I (IGF-I) to promote proteoglycans (PG) synthesis and viability [17]. Excess UPR activation can promote chondrocyte hypertrophy or apoptosis [15,16], thereby potentially accelerating OA progression.

UPR activation and function in OA pathogenesis are not well understood. Moreover, prior function analyses of XBP1 and CHOP in chondrocytes or chondrocytic cell lines have been limited to date [13,15,16]. Hence, we examined activation and function of the UPR in human and bovine knee articular chondrocytes, focusing on CHOP, and on biomechanical injury and responses to IL-1β, an inflammatory mediator in OA [1]. Since the anti-inflammatory metabolic super-regulator, AMP kinase (AMPK), limits activation of the UPR in vascular muscle cells [18], this study examined the effects of AMPK activity on the UPR in chondrocytes. AMPK inhibits pro-catabolic effects of IL-1β and TNFα, and conversely, IL-1β and TNFα induce decreased activity of AMPK [19]. Moreover, some drugs that are already used in the clinic for arthritis and other conditions activate AMPK. These include methotrexate, which activates AMPK by 5-aminoimidazole-4-carboxamide ribonucleotide (AICAR) generation, and the allosteric AMPK activators sodium salicylate, high dose aspirin, as well as metformin and some PPARγ activators [19-21].

## Methods

### Reagents

All chemical reagents were from Sigma-Aldrich (St Louis, MO, USA), unless otherwise indicated. AICAR, Compound C, and recombinant human cytokines were purchased from R&D Systems, Inc. (Minneapolis, MN, USA). For western blotting, antibodies to phospho-AMPKα (Thr172), total AMPKα GRP78 and CHOP were from Cell Signaling Technology, Inc. (Danvers, MA, USA). Antibodies to GRP78 and CHOP for immunohistochemistry (IHC), and to XBP1 for western blots, were from Abcam (Cambridge, MA, USA). Human CHOP, GRP78 and XBP1 siRNAs and control siRNAs were from Invitrogen (Life Technologies, Grand Island, NY, USA) and Santa Cruz Biotechnology (Santa Cruz, CA, USA).

### Studies of human knee articular chondrocytes

Studies were performed using institutionally reviewed and approved human subjects/ethics protocols by the VA, University of California San Diego, and Scripps Research Institute Human Subjects Institutional Review Boards. Human knee chondrocytes were isolated from autopsy donors as leftover de-identified material and with no interactions with subjects, and therefore with no informed consent required. The sources of cartilages were Lone tree tissue bank, Lone Tree, CO, USA, and South Texas Tissue Bank, San Antonio, TX, USA. Cartilages were graded macroscopically according to a modified Outerbridge scale, in which grade I was normal cartilage, grade II mild OA, grade III moderate OA, and grade IV severe OA [22]. Human chondrocytes were cultured in DMEM high-glucose medium with 10% FCS, 100 μg/ml streptomycin, and 100 IU/ml penicillin at 37°C, and no later than first-passage chondrocytes were used for all experiments [19]. Chondrocytes, whether bovine or human, were studied and compared from individual donors, in all experiments (that is, there was no pooling of cells from different donors). Unless otherwise indicated, chondrocytes were plated at 2.5 × 10^5^ cells per well in 250 μl of medium on the day before experimental treatment in 12-well plates.

### Immunohistochemistry for GRP78 and CHOP expression

Human knee cartilage sections were treated with 3% (vol/vol) H_2_O_2_ for 10 minutes, and were then blocked with 10% goat serum for 2 hours at room temperature. After washing with tris-buffered saline (TBS), rabbit antibodies to GRP78 (1:50 dilution) and CHOP (1:50 dilution) and the negative control rabbit immunoglobulin G (IgG) (1 μg/ml) were applied to the sections and incubated overnight at 4°C. Next, the sections were washed with TBS, incubated with biotinylated goat anti-rabbit IgG secondary antibody for 30 minutes, and then incubated for 30 minutes using the Histostain Plus kit (Invitrogen, Carlsbad, CA, USA). Finally, the sections were washed and incubated with 3,3′-diaminobenzidine (DAB) substrate for 2 to 5 minutes.

### Detection of XBP1 mRNA splicing by RT-PCR

Total RNA from the cells was isolated using RNeasy kit (Qiagen, Valencia, CA). Reverse transcription (RT) was carried out with 1 μg RNA using Transcriptor First Strand cDNA Synthesis kit (Roche, South San Francisco, CA). RT-PCR to determine the unspliced and spliced isoforms of XBP1-employed sequences of human XBP1 primers TTACGAGAGAAAACTCATGGCC (forward) and GGGTCCAAGTTGTCCAGAATGC (reverse), with sizes of PCR products 289 bp for unspliced XBP1 (XBP1u), and 263 bp for spliced XBP1 (XBP1s).

### Chondrocytes subjected to biomechanical injury

Bovine articular chondrocytes, isolated from mature cow knees, as described [19], were embedded in 2% alginate discs with 6 mm diameter and 3 mm height, as described [23]. In brief, chondrocyte-alginate constructs were then cultured in DMEM containing 10% FBS and 1% penicillin-streptomycin, in a 37°C, 5% CO2 incubator for 3 to 4 weeks to allow for extracellular matrix production before application of biomechanical injury [21]. The injury protocol was via a custom-made mechanical compression apparatus housed inside a 37°C, 5% CO2 incubator. The sub-lethal injury condition, defined as no immediate but delayed cell death to about 25% of cells after injury, determined *in situ* by Live/Dead cell viability assay (Invitrogen, CA), involved continuous dynamic unconfined compression at 24% strain, with 12% amplitude at 0.5 Hz for 16 hours [23]. Alginate-chondrocyte constructs were then cultured and analyzed at 0, 1, and 2 days after injury. Chondrocytes were isolated from the constructs by dissolution of alginate using a 50-mM ethylenediaminetetraacetic acid (EDTA)/PBS solution. To test the effect of AICAR, the alginate-chondrocyte constructs were pre-treated with AICAR (1 mM) for 24 hours, and then subjected to mechanical injury and cultured for 2 days.

### Knockdown of CHOP, GRP78 or XBP1, and overexpression of CHOP in human knee articular chondrocytes

Normal, cultured, human knee articular chondrocytes (passage 1) were transfected with siRNAs of CHOP, GRP78 or XBP1 and the non-target controls, or transfected with CHOP driven by the CMV promoter in pCMV6-CHOP (Origene, Rockville, MD), or the vector plasmid control, using XtremeGene siRNA or Xtreme DNA transfection reagents (Roche), respectively. Expression of CHOP, GRP78 and XBP1 was examined by SDS-PAGE/western blot.

### Measurement of nitric oxide generation, matrix metalloproteinase (MMP)-3 release, oxidative stress and apoptosis in chondrocytes

Conditioned media were analyzed for NO and MMP-3 by Griess reaction and western blot, respectively, as described [19,23]. Chondrocyte oxidative stress and apoptosis were assessed by Mitosox Red and annexin V-fluorescein isothiocyanate (FITC) staining for mitochondrial superoxide generation and apoptotic cells, and presented as percentage of cells stained positively for Mitosox Red and annexin V, respectively, relative to total cell numbers determined by Hoechst 33342 nuclear staining.

### Statistical analyses

All data were uniformly expressed as mean ± SD. Statistical analyses were performed by two-way analysis of variance (ANOVA) with Bonferroni post-hoc test using GraphPad PRISM 5. *P*-values less than 0.05 were considered significant.

## Results

### Chondrocyte CHOP expression in human knee OA and in cultured chondrocyte responses to IL-1β and biomechanical injury

**Figure 1:**
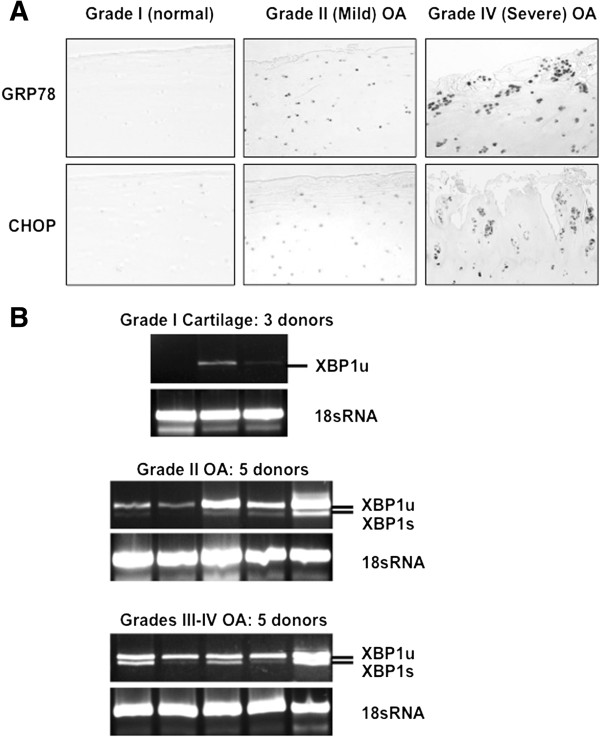
**Increased 78-kDa glucose-regulated protein (GRP78), C/EBP binding protein (CHOP), and X-box binding proteins (XBP1s) in human osteoarthritis (OA) chondrocytes. (A)** Immunohistochemistry revealed expression of GRP78 and CHOP in human normal knee (grade I) and knee OA cartilage (mild and severe OA, grades II and IV, respectively). Data representative of a total five normal donors, and of six different donors each with mild and severe knee OA. **(B)** Reverse transcription (RT)-PCR was performed to assess XBP1 splicing at mRNA level in passage-1 human knee chondrocytes ranging from normal to severe OA, studying 1 × 10^6^ cells, as described (Methods), in normal (grade I) and OA cells. Alternative splicing of XBP1 mRNA (248 bp), and increased XBP1 mRNA, were only seen in OA samples. The spliced XBP1 product XBP1s was 26 bp shorter (lower band), and was seen in chondrocytes of four of the five grade-II knee OA donors, and all five grade III-IV samples.

Both CHOP and GRP78 expression, as detected by IHC, increased markedly in advanced OA knee cartilage *in situ*, particularly in chondrocyte clusters (Figure 1A). Confirming UPR activation [13,14] in more advanced OA, we detected UPR-specific XBP1s mRNA alternative splicing in chondrocytes from four out of five different donors with human knee grade II OA, and in all of five different donors with grades III-IV knee OA, but not in any of the three different normal human knee cartilage chondrocyte donors (Figure 1B). XBP1u, which normally has a very short half-life (6 minutes), also was readily detected in chondrocytes from each of the ten donors with grades II-IV knee OA, whereas XBP1u was inconsistently detected in the chondrocytes of the three normal knee-cartilage donors (Figure 1B).

**Figure 2:**
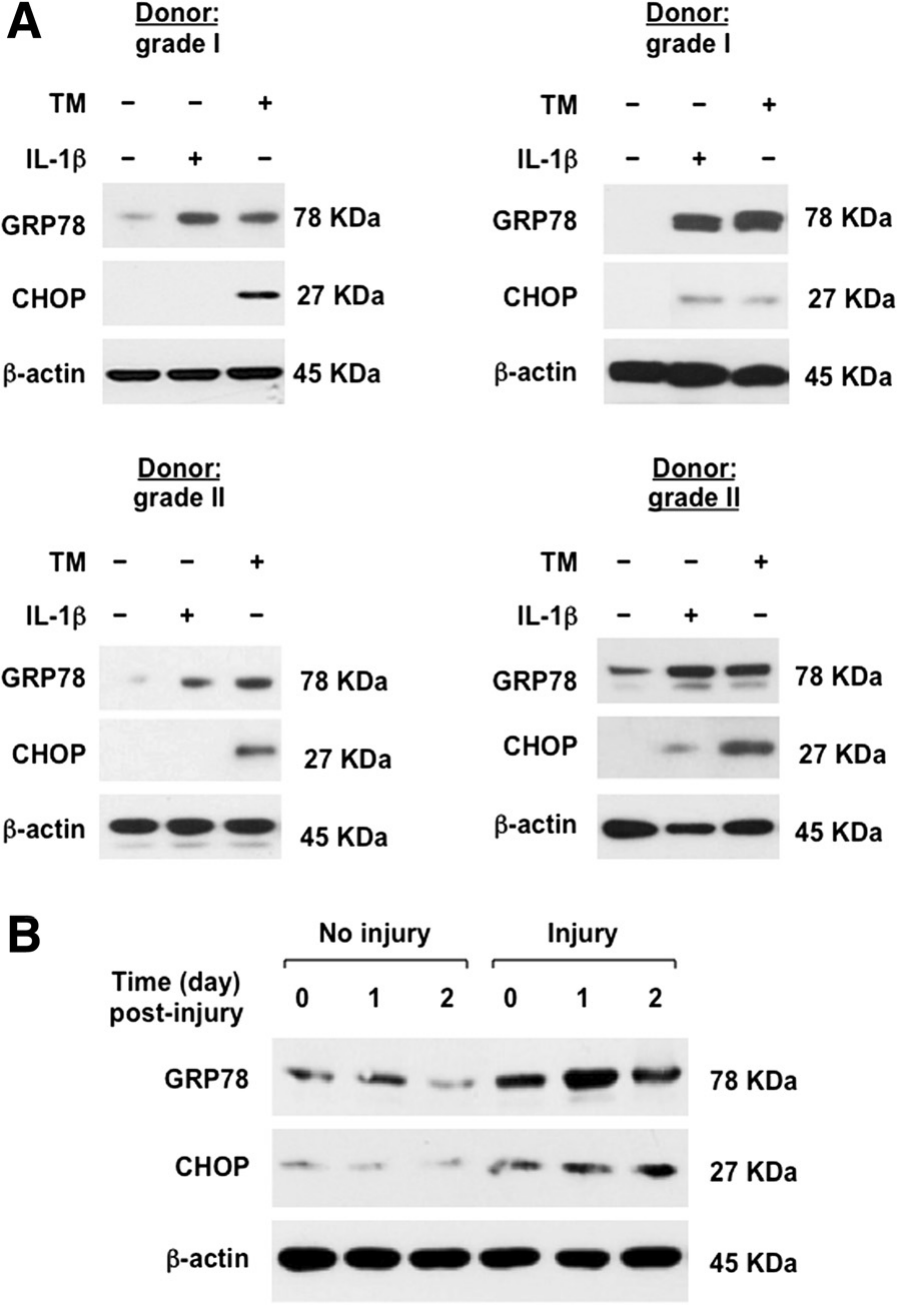
**Effects of IL-1β and biomechanical injury on 78-kDa glucose-regulated protein (GRP78) and C/EBP binding protein (CHOP) expression in chondrocytes. (A)** SDS-PAGE/western blot analysis was performed to examine expression of GRP78 and CHOP in passage-1 human knee chondrocytes that we treated with IL-1β (10 ng/ml) and the positive control UPR inducer tunicamycin (TM, 2.5 μg/ml), for 18 hours. In all, 13 different donors (3 grade I, 5 between grade I and II, and 5 grade II) were studied; results of 4 donors are shown. See Table 1 for donor-grading information. CHOP induction by IL-1β was detected in only one grade I and one grade II knee cartilage donor of 13. **(B)** Primary bovine-knee chondrocytes embedded in alginate were subjected to mechanical injury, and cell protein extracted as described (Methods). Data representative of three individual experiments.

Next, we studied human knee articular chondrocytes stimulated with IL-1β, and the positive control UPR inducer tunicamycin (TM) (Figure 2A). Studying protein expression by SDS/PAGE-western blotting, we detected induction of GRP78 by IL-1β, in all of the 13 different human knee donor articular chondrocyte lysates, whereas we did not detect increased CHOP expression in response to IL-1β in 11 of the 13 different knee-cartilage donor chondrocytes (Figure 2A; Table 1). By comparison, the sub-lethal biomechanical injury protocol [23] induced increased expression of both CHOP and GRP78 in bovine-knee articular chondrocytes embedded in alginate *in vitro* (Figure 2B).

**Table 1:** **Cartilage-donor grading and CHOP results for Figure**2**, panel A**

**Cartilage grade**	**CHOP induction by IL-1β**
I	No
II	No
II	No
II	No
I-II	No
II	No
I-II	No
I-II	No
I-II	No
I-II	No
I	Yes
I	No
II	Yes

Chop, C/EBP binding protein.

### Effects of CHOP, XBP1s, and GRP78 gain of function and loss of function in chondrocytes

**Figure 3:**
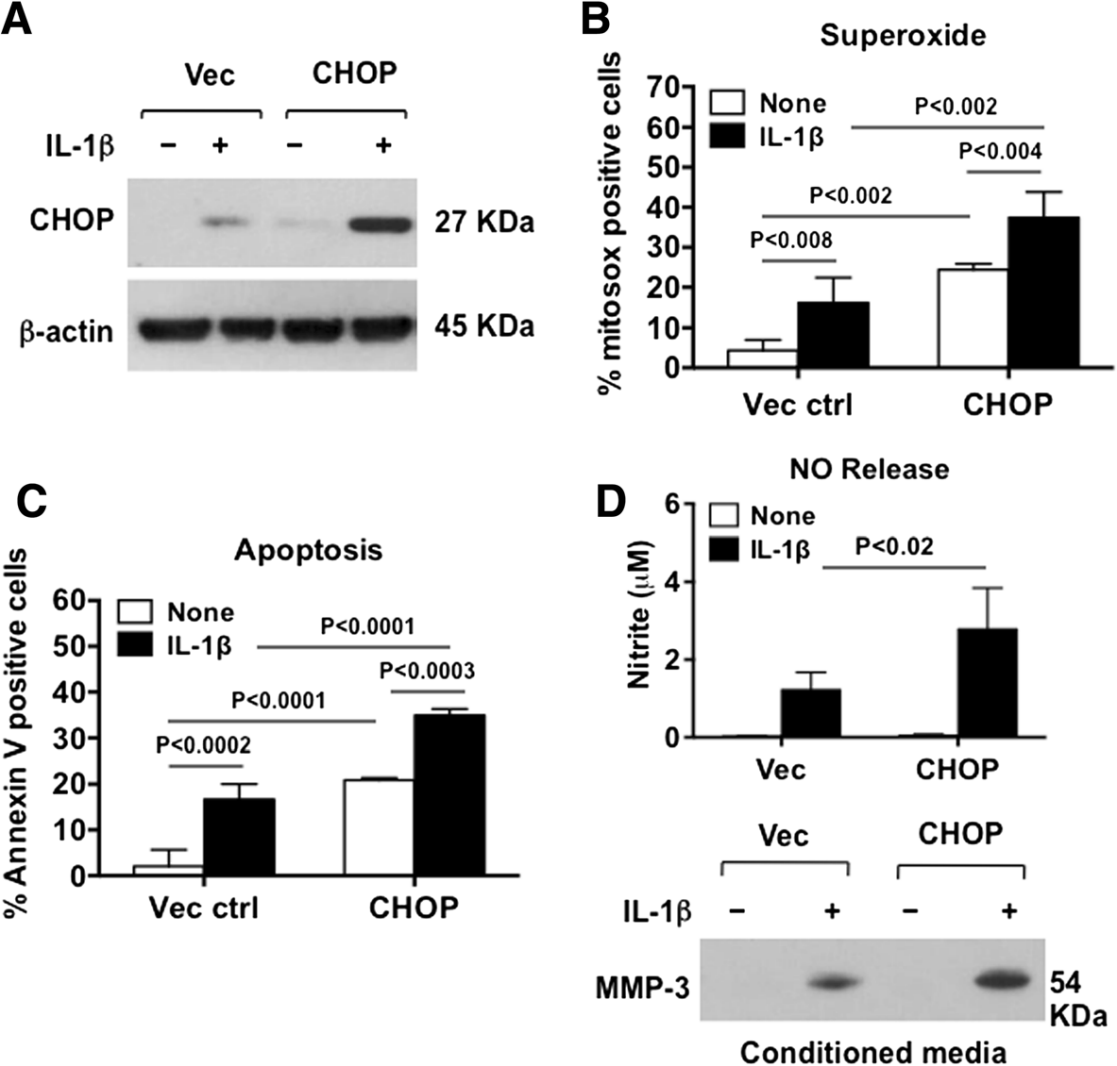
**Effects of gain of function of C/EBP binding protein (CHOP) on release of nitric oxide (NO) and matrix metalloproteinase-3 (MMP-3), and on oxidative stress and apoptosis in chondrocytes.** Cultured human knee chondrocytes (passage 1) were transfected with pCMV6-hCHOP and the vector control (Vec ctrl) for 48 hours, then stimulated with IL-1β (10 ng/ml) for 18 hours **(A**, **D)** or 6 hours **(B**, **C). (A)** Expression of CHOP was examined by western blot. Superoxide generation and apoptosis were determined by percentage of cells stained positively for Mitosox Red **(B)** and annexin V **(C)**. Release of NO and MMP-3 **(D)** were assessed by Griess reaction and western blot, respectively, in conditioned media. Data representative of experiments on three different knee chondrocyte donors.

We focused on CHOP gain of function, via transfection of CHOP cDNA in human chondrocytes (Figure 3A). We gauged the effects of enhanced CHOP expression on chondrocyte function with and without IL-1β stimulation (Figure 3B-D). In these studies, IL-1β induced CHOP expression in the five donors tested after transfection, even in samples where there was no induction of CHOP by IL-1β without transfection (not shown). Because this was assumed to reflect compound cell stress of the transfection and IL-1β treatment, this was not further evaluated here. We observed that CHOP gain of function, by itself, induced superoxide production and chondrocyte apoptosis, and effects of CHOP and IL-1β on superoxide and apoptosis were additive (Figure 3B-C). In contrast, CHOP gain of function was not sufficient to induce NO and MMP-3 release, but CHOP gain of function significantly potentiated the capacity of IL-1β to induce NO and MMP-3 release (Figure 3D).

**Figure 4:**
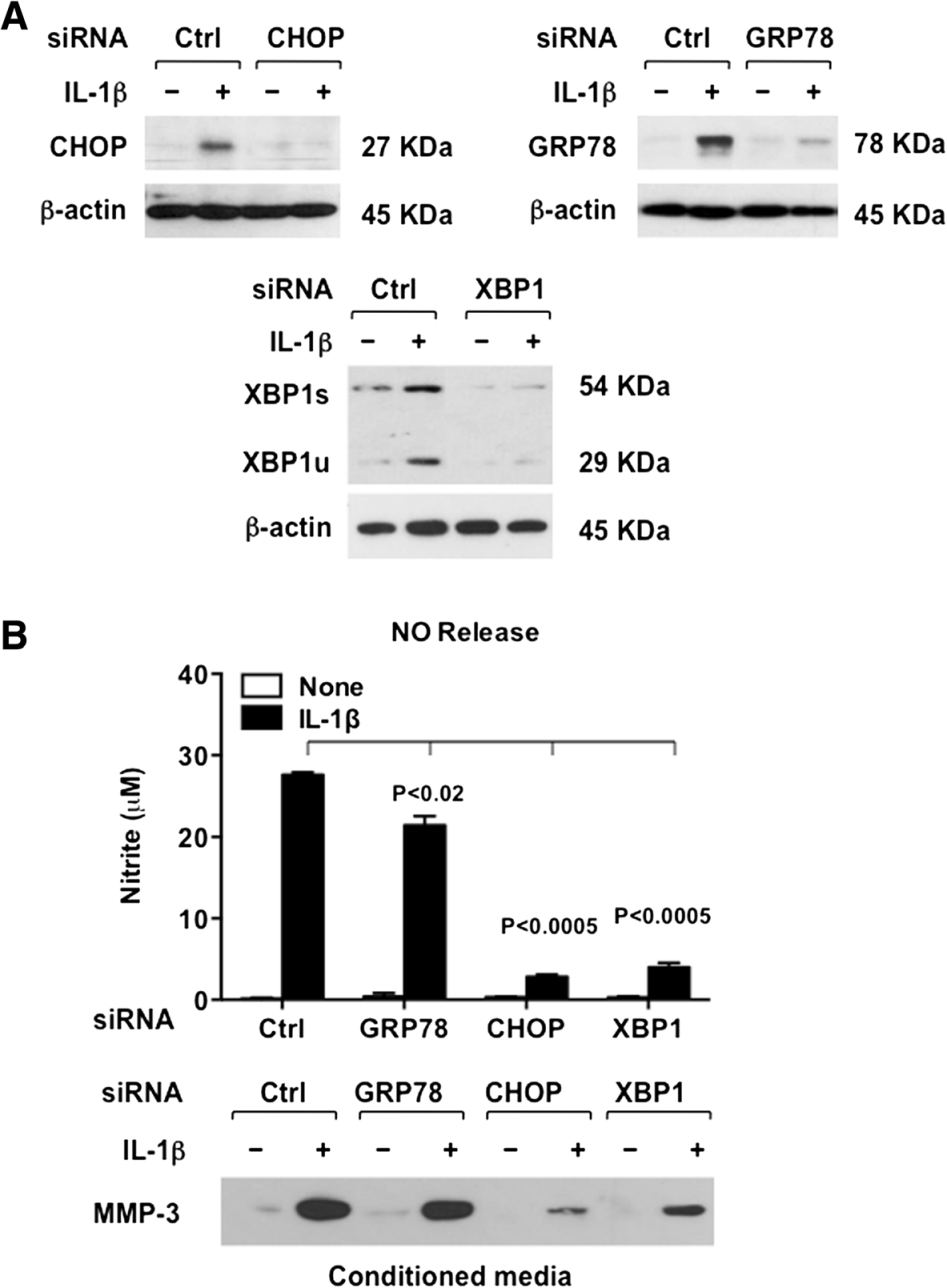
**Effects of knockdown of C/EBP binding protein CHOP, X-box binding protein (XBP1), and 78-kDa glucose-regulated protein (GRP78) on release of nitric oxide (NO) and matrix metalloproteinase-3 (MMP-3) in response to IL-1β in chondrocytes. (A)** Cultured human knee chondrocytes (passage 1) were transfected with siRNAs of CHOP, GRP78 and XBP1 and respective control (ctrl) siRNAs. Knockdown of expression of CHOP, GRP78 and XBP1 was confirmed by western blot. **(B)** Chondrocytes with validated knockdown of CHOP, GRP78 and XBP1 were treated with IL-1β (10 ng/ml) for 18 hours, and release of NO and MMP-3 determined. Data representative of experiments on three different human knee chondrocyte donors.

We next focused on defining and comparing the need for CHOP versus the UPR mediators XBP1s and GRP78, in NO and MMP-3 release, via a loss-of-function approach we first validated *in vitro* (Figure 4A). CHOP and XBP1 siRNA knockdown blunted NO release by >80% (P <0.0005) in response to IL-1β, and also attenuated MMP-3 release (Figure 4B). GRP78 siRNA knockdown, by comparison, exerted minimal effects on both of these pro-catabolic responses (Figure 4B).

### Reciprocal regulation of CHOP and AMPK in injured chondrocytes

**Figure 5:**
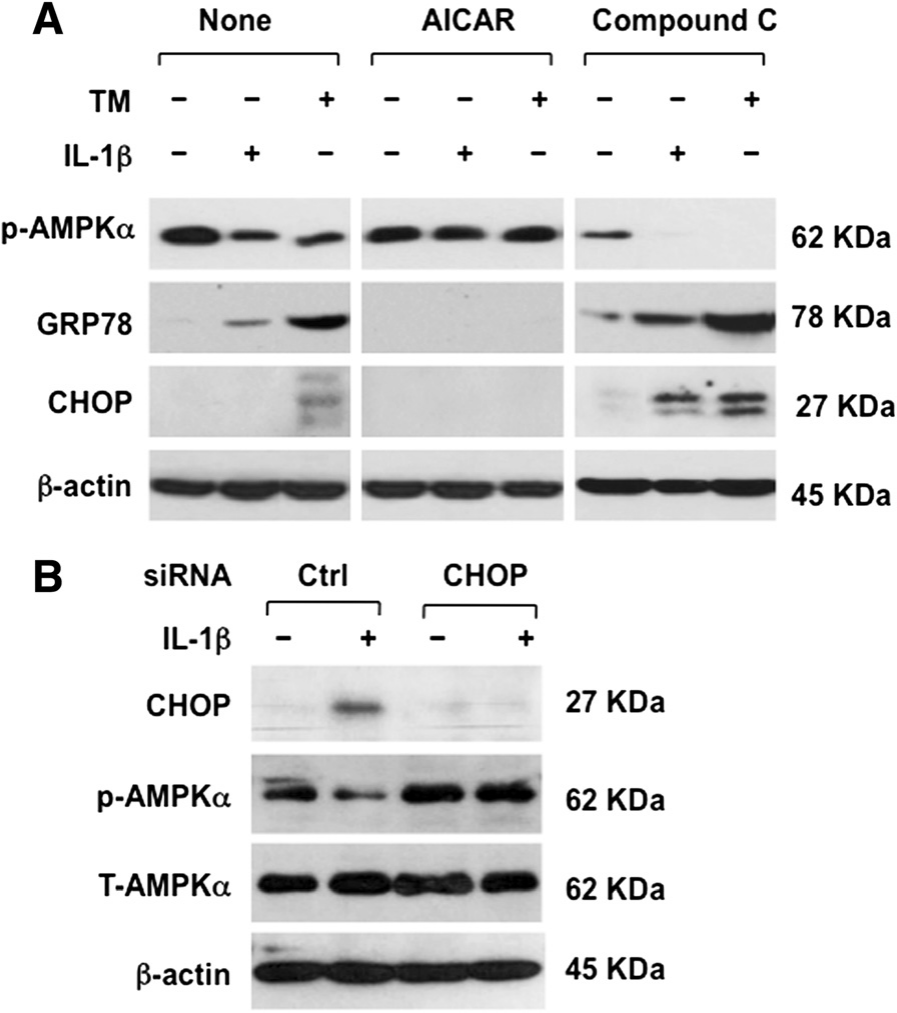
**Reciprocal regulation of AMP-activated protein kinase (AMPK) activity and C/EBP binding protein (CHOP) expression in chondrocytes. (A)** Normal bovine-knee chondrocytes (passage 1) were pre-treated with a pharmacological AMPK activator 5-aminoimidazole-4-carboxamide ribonucleotide (AICAR) (1 mM) or a pharmacological AMPK inhibitor compound C (10 μM) for 1 hour before stimulation with IL-1β (10 ng/ml) for 18 hours. AMPK activity (threonine phosphorylation of AMPKα) and total AMPKα were examined by SDS-PAGE/western blot. **(B)** CHOP siRNA knockdown in first-passage human knee chondrocytes suppressed the capacity of IL-1β (10 ng/ml) to promote de-phosphorylation of AMPKα. Data in panel **A** representative of three individual experiments with different bovine-chondrocyte donor knees, and in panel **B** representative of separate experiments on three different human knee chondrocyte donors.

AMPK is known to limit UPR activation in cells other than chondrocytes [18]. Hence, we next studied effects of AICAR, an AMPK activator. AICAR blunted the capacity of both IL-1β and tunicamycin to induce GRP78 in bovine chondrocytes *in vitro*, under conditions where AICAR blunted tunicamycin-induced CHOP expression (Figure 5A). Conversely, the AMPK inhibitor, Compound C, potentiated IL-1β-induced CHOP and GRP78 expression in chondrocytes (Figure 5A). CHOP siRNA knockdown reduced the capacity of IL-1β to suppress levels of active AMPK (p-AMPK), as assessed by AMPK threonine phosphorylation, and standardized to total AMPK (T-AMPK) (Figure 5B).

**Figure 6:**
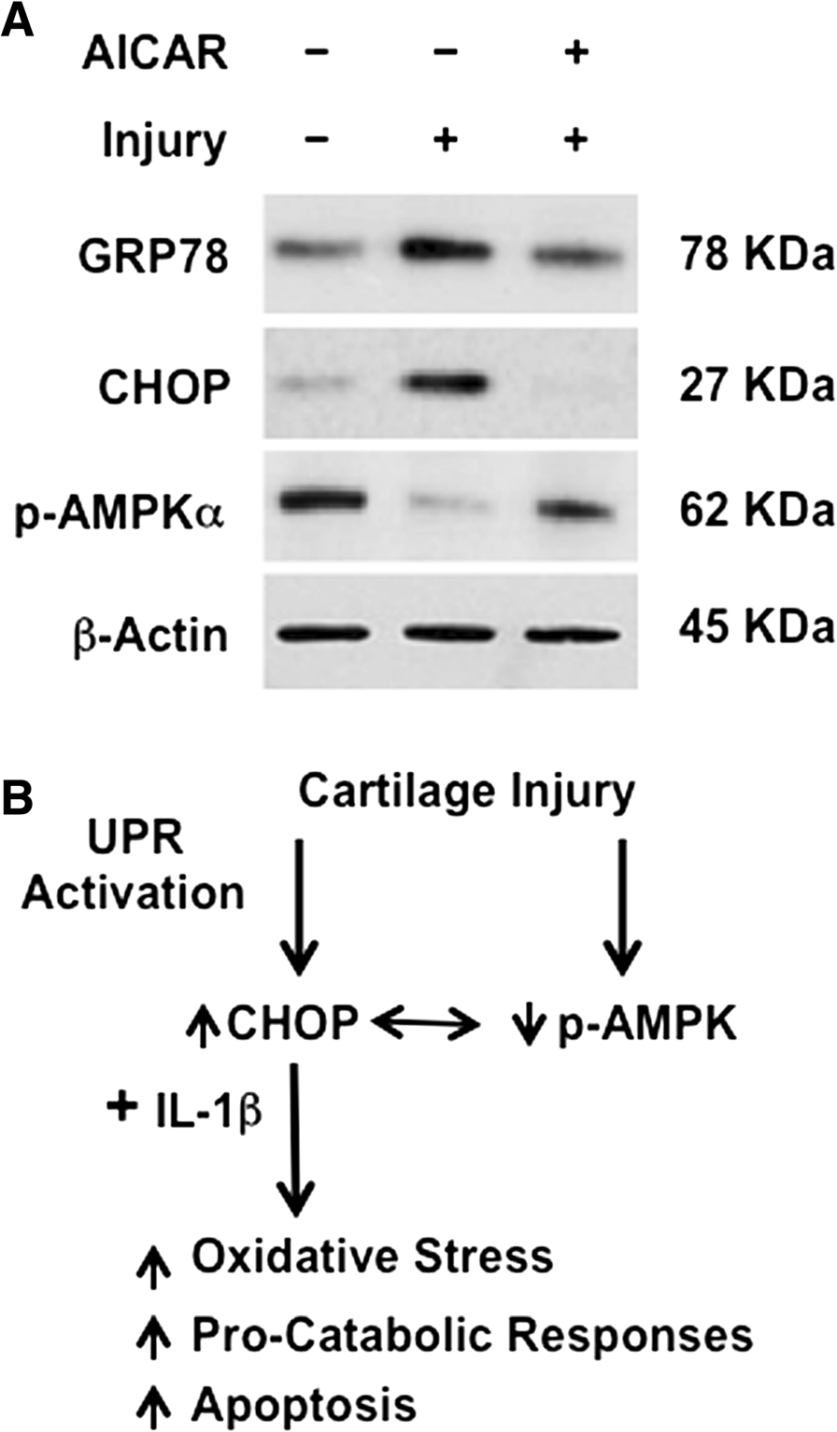
**Chondrocyte AMP-activated protein kinase (AMPK) activity modulates the unfolded protein response (UPR) and completes the model illustrated here. (A)** Primary bovine-knee chondrocytes embedded in alginate were pre-treated with activator 5-aminoimidazole-4-carboxamide ribonucleotide (AICAR) (1 mM) for 24 hours before being subjected to mechanical injury as described (Methods). Expression of 78-kDa glucose-regulated protein (GRP78) and C/EBP Binding protein (CHOP) was examined by western blot. Data are representative of three individual experiments. **(B)** In this model, biomechanical injury induces decreased AMPKα activity (via dephosphorylation) and also induces CHOP expression in chondrocytes. AMPK activity limits CHOP expression. Conversely, induction of decrease in AMPK activity plays a major role in the capacity of IL-1β to induce CHOP expression. CHOP mediates oxidative stress and apoptosis in chondrocytes. CHOP also potentiates the capacity of IL-1β to promote certain pro-catabolic responses in chondrocytes.

Last, we assessed for hypothesized reciprocal regulation of CHOP and AMPK activity in biomechanical injury of the normal bovine-knee chondrocyte constructs in alginate. We first observed that biomechanical injury induced CHOP and GRP78, under conditions where we confirmed [23] decreased active AMPK (Figure 6A). Pharmacologic AMPK activation blunted injury-induced CHOP expression, and inhibited injury-induced GRP78 expression (Figure 6A). Overall results of this study are summarized in the schematic of Figure 6B.

## Discussion

Abnormal function of proteostasis mechanisms mediates changes in cell differentiation and loss of viability, and thereby is held to contribute to progressive tissue damage in aging, and metabolic and inflammatory diseases [2-5,24,25]. Here, we provided evidence for UPR activation in advanced human knee OA articular chondrocytes, including particularly robust CHOP and GRP78 expression in chondrocyte clusters *in situ*, and UPR-specific XBP1s generation in cultured chondrocytes.

CHOP has a short half-life in many cells [9,10], a prime example of intrinsic instability of UPR mediators that promote apoptosis, in comparison to others that facilitate protein folding and cell redox regulation in order to promote cell survival in response to stress [26]. Here, we demonstrated that biomechanical injury rapidly induced robust and sustained CHOP and GRP78 expression in cultured chondrocytes. In contrast, IL-1β consistently induced GRP78 without inducing CHOP in chondrocytes *in vitro*. Compound cell stresses inherent in biomechanical injury may have accounted for this distinction in UPR response, relative to the response to IL-1β in isolation. Toll-like receptor (TLR)4 signaling adaptively suppresses CHOP expression to help keep cells viable when sensing danger signals [10]. As there are broad interactions between TLR4 and IL-1 signaling, potential differences in TLR4 signaling between biomechanical injury and IL-1 signaling could hold clues to variability in chondrocyte UPR responses, such as those seen in this study. Because chondrocytes from two of thirteen different human donors responded to IL-1β with robust CHOP expression, it remains to be determined if increased CHOP expression in cartilage is a biomarker for increased progression in evolving OA. This is particularly pertinent, since CHOP appeared necessary for maximal NO and MMP-3 release by chondrocytes in our siRNA studies. Moreover, we observed that CHOP gain-of-function increased chondrocyte superoxide generation and apoptotic responses to IL-1β.

Importantly, CHOP acted at least in part by promoting decreased AMPK activity, and AMPK activity limited CHOP expression in chondrocytes in response to either biomechanical injury or IL-1β. AMPK has been recently discovered to limit UPR activation in the vascular system *in vivo*[18,27]. Moreover, we have recently reported decreased phosphorylation of AMPKα in human knee OA chondrocytes [19]. AMPK inhibits pro-catabolic effects of IL-1β and TNFα, and conversely, IL-1β and TNFα induce decreased activity of AMPK, but these effects can be prevented by pharmacologic activation of AMPK [19], as done here using AICAR. Hence, it is translationally pertinent for OA that AMPK is activated by several drugs already in the clinic for arthritis and other conditions.

This study provided further evidence [13] for UPR-specific XBP1 activation, assessed via XBP1s generation, in moderate to severe human knee OA. XBP1s promotes ER degradation of aberrant proteins [4], and amplifies TLR2/4-driven innate immune cytotoxic and inflammatory responses [6,7], but paradoxically XBP1s inhibits NF-κB, and exerts anti-inflammatory effects [8,28]. Here, we observed that XBP1s has at least one noxious function in chondrocytes, since XBP1 siRNA knockdown robustly inhibited NO and MMP-3 release in response to IL-1β. XBP1 could exert some chondroprotective effects, since the IRE1alpha-XBP1 cascade can promote or inhibit autophagy, depending on cell type and biologic context [4,29,30]. Furthermore, chondrocytes *in situ* are in a hypoxic milieu, and in hypoxic cells, XBP1 is a major survival factor [31].

Increased expression of the chaperone GRP78 is a biomarker of increased ER stress [2-5], and was confirmed [14] to be increased in human knee OA in this study. GRP78 normally dampens the UPR by binding to and thereby limiting signaling of all three principal UPR ER transmembrane-expressed molecular signaling cascade initiators [2-5]. An important result in this study, consistent with this UPR physiology, was that GRP78 loss of function, unlike the case for CHOP and XBP1 loss of function, had limited influence on the capacity of IL-1β to promote chondrocyte NO and MMP-3 release. However, we did not examine additional pro-catabolic responses.

This study focused on the functional consequences of increased UPR activity, including CHOP expression, in an examined group of primarily normal donor chondrocytes. Clearly, excess UPR activation triggers cartilage developmental pathology *in vivo*[11,12]. Whether effects of excess UPR activity seen here in cultured chondrocytes contribute to OA in aging cartilage is a pertinent question, in part because decreased AMPKα activity is linked with aging in knee cartilage [19,23]. Moreover, UPR activation is linked with multiple degenerative tissue diseases of aging, mediated by oxidative damage to UPR mediators and accumulation of misfolded proteins [5]. Specific UPR alterations with aging have thus far primarily been described in neurodegenerative and liver disease, decreased expression and function of GRP78, and conversely, increased sensitivity of caspase-12 and CHOP induction in brain [5].

An ER stress inhibitor *in vivo* reduced apoptosis and thinning of mandibular cartilage observed after mechanical stress loading [32]. One limitation of our study is that we did not study the effects of CHOP pharmacologic inhibition or CHOP knockout on experimental OA triggered by biomechanical instability in mice. In this context, CHOP^-/-^ mice are grossly normal, and breed and develop their skeleton in a grossly normal way by gross morphology. Though targeting increased CHOP expression has chondroprotective potential in OA, such an approach directly targeted to CHOP would need to avoid CHOP deficiency, since CHOP knockout mice have defective osteoblast maturation and a decreased rate of bone mineral formation [33-36]. As such, distinguishing the effects of CHOP inhibition on OA *in vivo* would need to pay heed to effects of CHOP on bone, and other joint tissues, not simply articular cartilage. Other limitations of this study include that we did not characterize protein misfolding on chondrocytes, and did not address mediators in all cascades of the UPR. In addition, the expression analyses of XBP1 in human knee OA were limited to qualitative PCR in methodology, and that we did not explore what the potential effects are in OA of XBP1u, the expression of which was more consistently detected in chondrocytes from human knee OA than normal cartilages.

## Conclusions

Both biomechanical injury and IL-1β stimulate UPR activation in chondrocytes. Moreover, CHOP drives chondrocyte pro-catabolic responses to IL-1β, mediated by regulation of AMPK activity. Our results suggest that pharmacologic AMPK activation could have therapeutic potential in OA, partly by limiting excess CHOP induction in response to injury.

## Abbreviations

AICAR: 5-aminoimidazole-4-carboxamide ribonucleotide; AMPK: AMP-activated protein kinase; ATF6: Activating transcription factor 6; bp: Base pairs; CHOP: C/EBP binding protein; DMEM: Dulbecco’s modified Eagle’s medium; eIF2A: Eukaryotic initiation factor 2A; ER: Endoplasmic reticulum; ERAD: ER degradation; FBS: Fetal bovine serum; FCS: Fetal calf serum; FITC: Fluorescein isothiocyanate; GADD34: Growth arrest and DNA damage-inducible protein 34; GRP78: 78-kDa glucose-regulated protein; IgG: Immunoglobulin G; IHC: Immunohistochemistry; IL: Interleukin; IRE1: Inositol-requiring enzyme 1; MMP: Matrix metalloproteinase; NO: Nitric oxide; OA: Osteoarthritis; p-AMPK: Phosphorylated (active) AMPK; PERK: Pancreatic ER kinase; T-AMPK: Total AMPK; TBS: Tris-buffered saline; TLR: Toll-like receptor; TNF: Tumor necrosis factor; UPR: Unfolded protein response; XBP1: x-box binding protein; XBP1s: Spliced XBP1; XBP1u: Unspliced XBP1.

## Competing interests

Dr Liu-Bryan holds a patent on use of AMPK activation for treatment of OA, which is subject matter related to manuscript.

## Authors’ contributions

MH worked to interpret and perform UPR mediators, develop and design the biomechanics experiments and general design of UPR studies analyzing expression levels, provided initial input and final critical comments on the writing of the manuscript, and approved the final version. FP performed and interpreted the biomechanics studies, provided critical comments on the writing of the manuscript, and approved the final version. ML oversaw collection and grading of human cartilages and chondrocytes, and helped with critical input in design and interpretation of the studies, and critical comments on writing of the manuscript, and approved the final version. RT conceived the experimental plan, oversaw design and execution of the experiments, interpreted all the data, and played the primary role in writing the manuscript, and approved the final version. RL-B directly supervised execution of all the experiments, designed, performed and interpreted all the CHOP expression and function studies, and played a major role in writing of the manuscript, and approved the final version. All authors read and approved the final manuscript.
